# Supplementary material for: SARS-CoV-2 intra-host variation shows evidence of transmission and convergent evolution in a university surveillance cohort
Source: Microb Genom. 2026 Sep 7;12(9):001784. doi: 10.1099/mgen.0.001784 (PMC13549476; doi:10.1099/mgen.0.001784)
Supplement: Supplementary file 1 [file mgen-12-01784-s001.pdf]

Supplementary Material

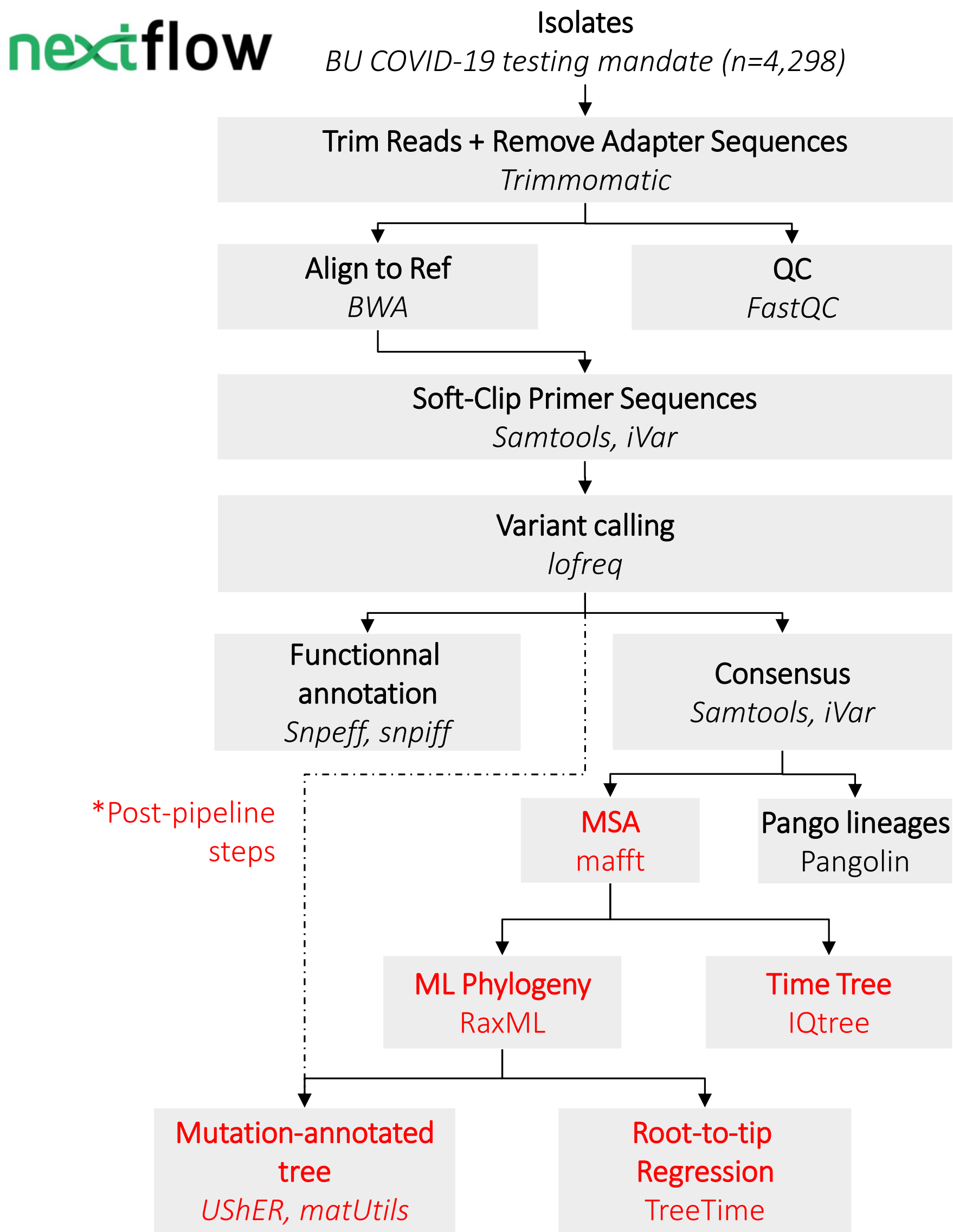

Figure S1. Flowchart of the bioinformatic processing steps. The code is for these processing steps is publically available on GitHub at: https://github.com/Leacavalli/Sars-cov-2-Intrahost-Variation.

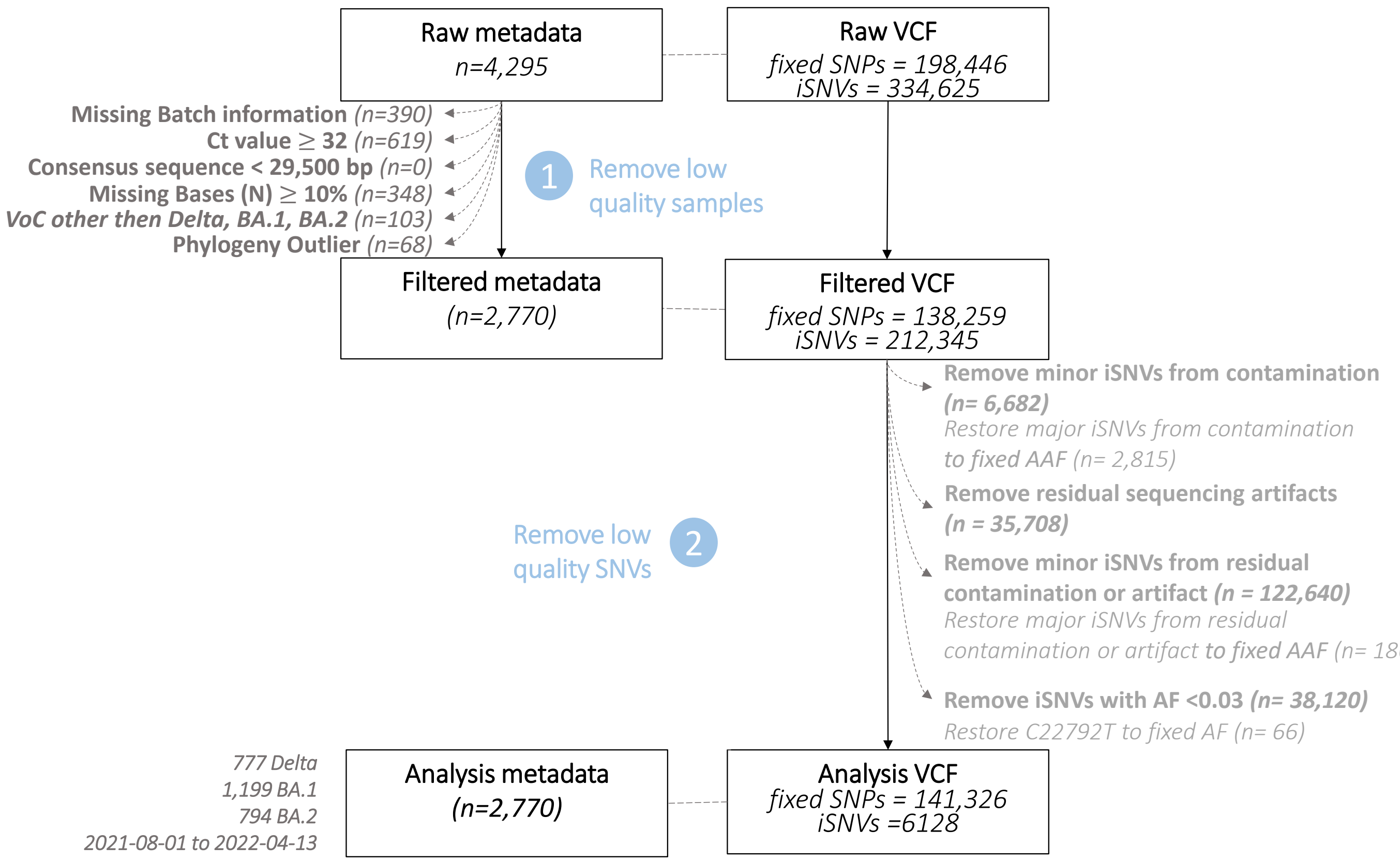

Figure S2. Flowchart of the data filtering and cleaning process. The metadata files are depicted on the left, while the multi-sample VCF file containing single nucleotides variant calls for all our isolates is shown on the right. The data cleaning process occurs in two stages: (1) The exclusion of low quality samples, and (2) The exclusion of low quality and false-positive iSNVs.

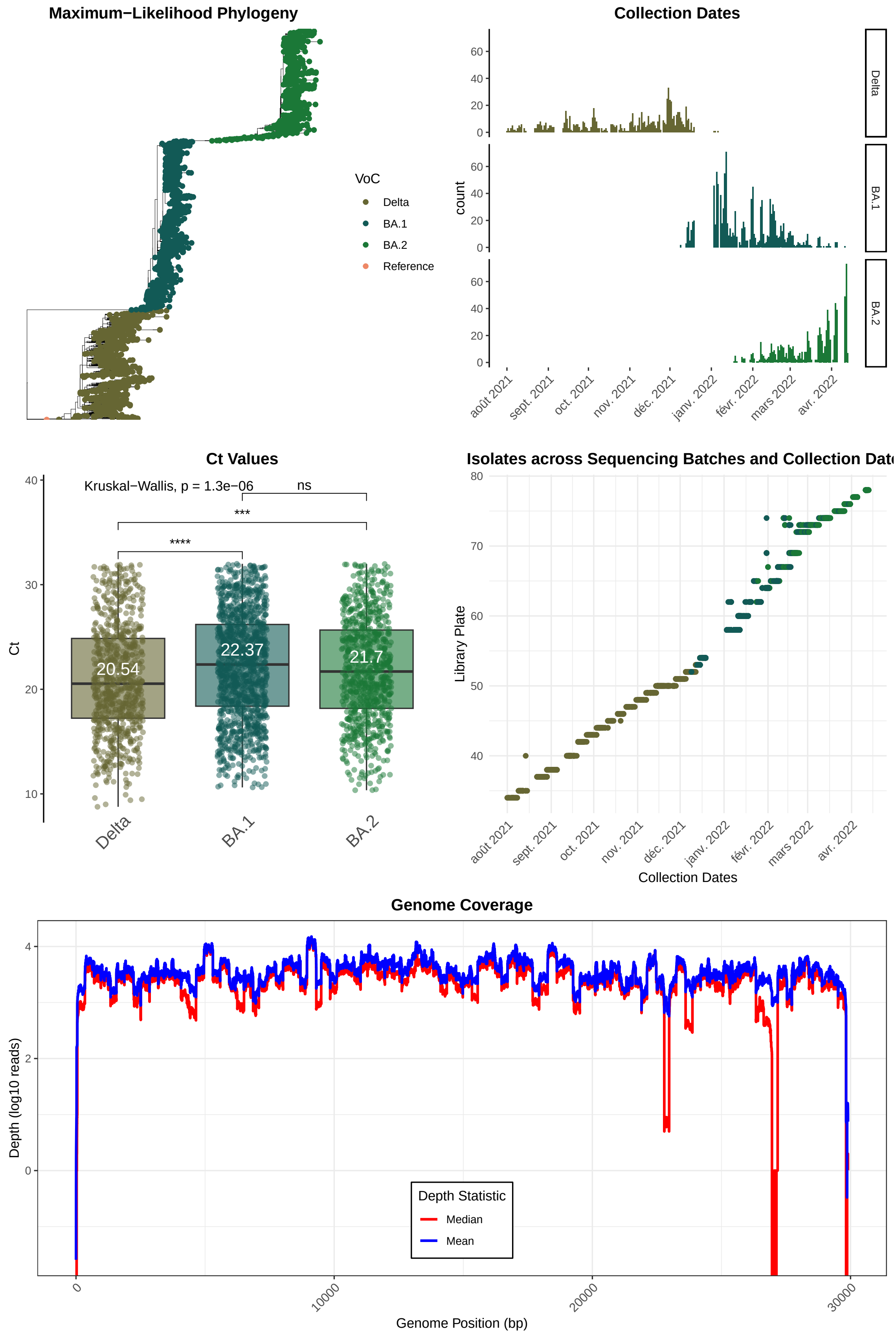

Figure S3. Sample characteristics. Consistent with previous reports, Delta isolates exhibit significantly smaller Ct values compared with BA.1 and BA.2 isolates, indicating higher viral loads.

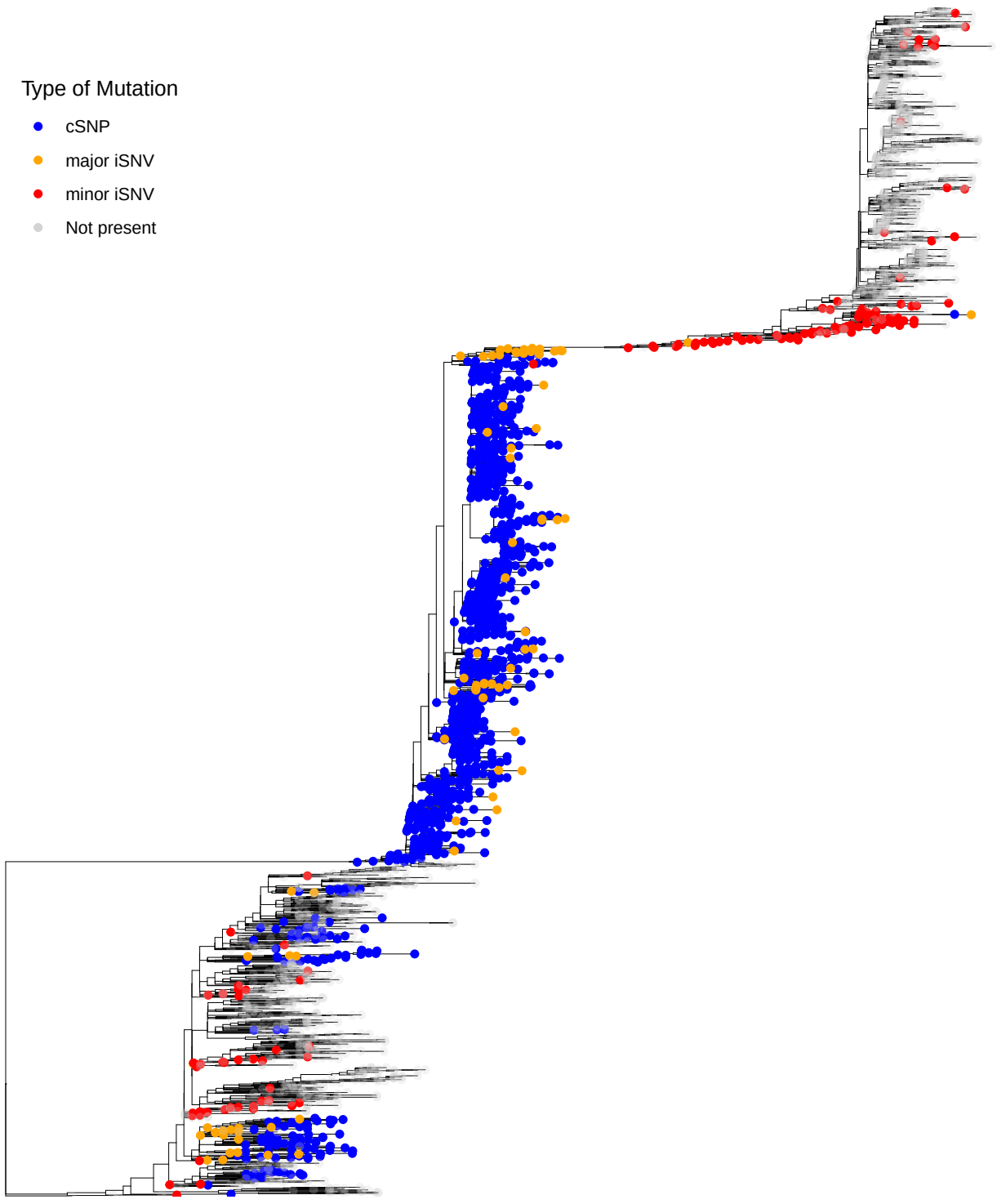

(a) Phylogenetic distribution of C21846T.

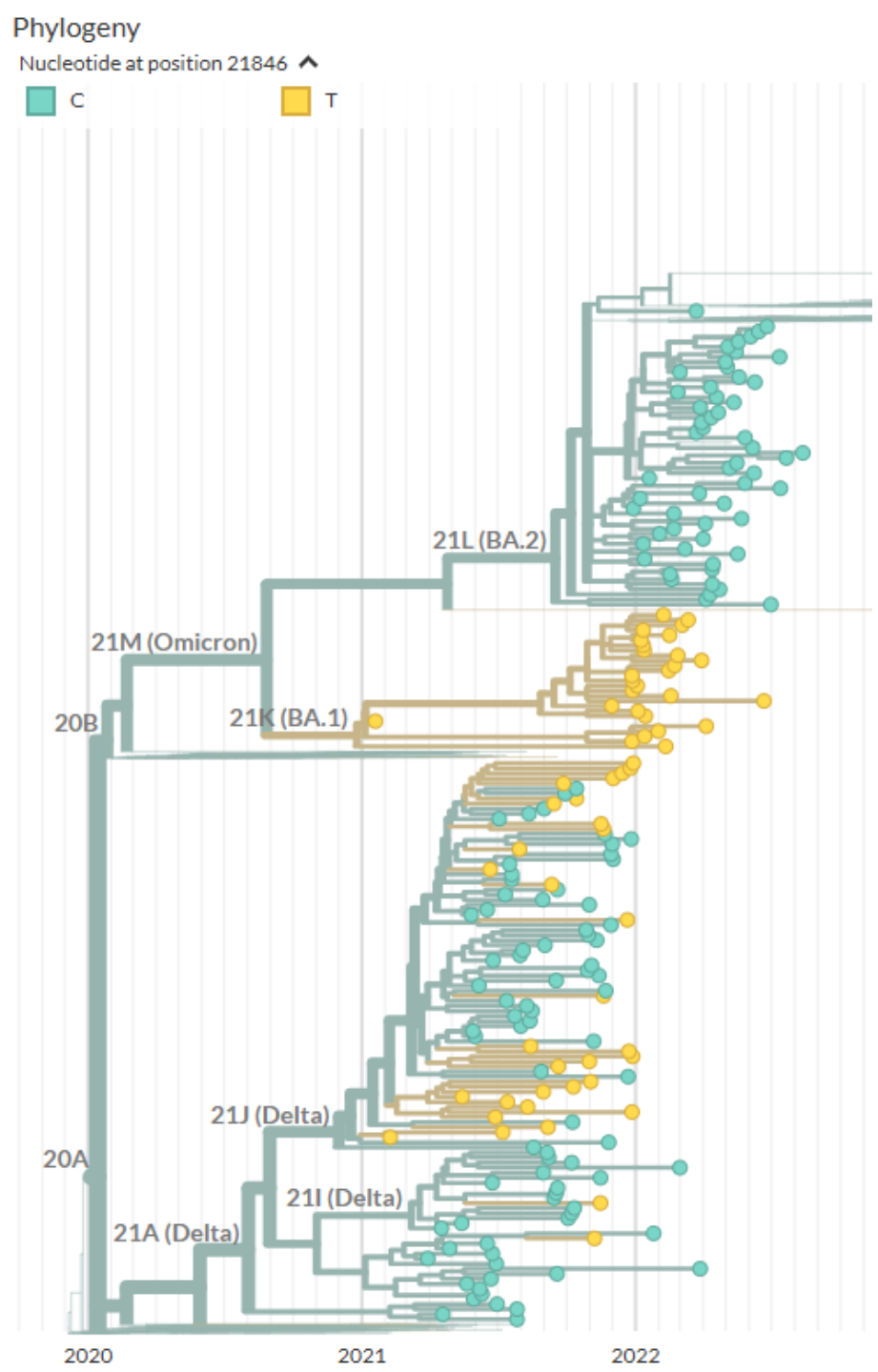

(b) Consensus allele at position 21,846 in GISAID (Nextstrain screenshot).

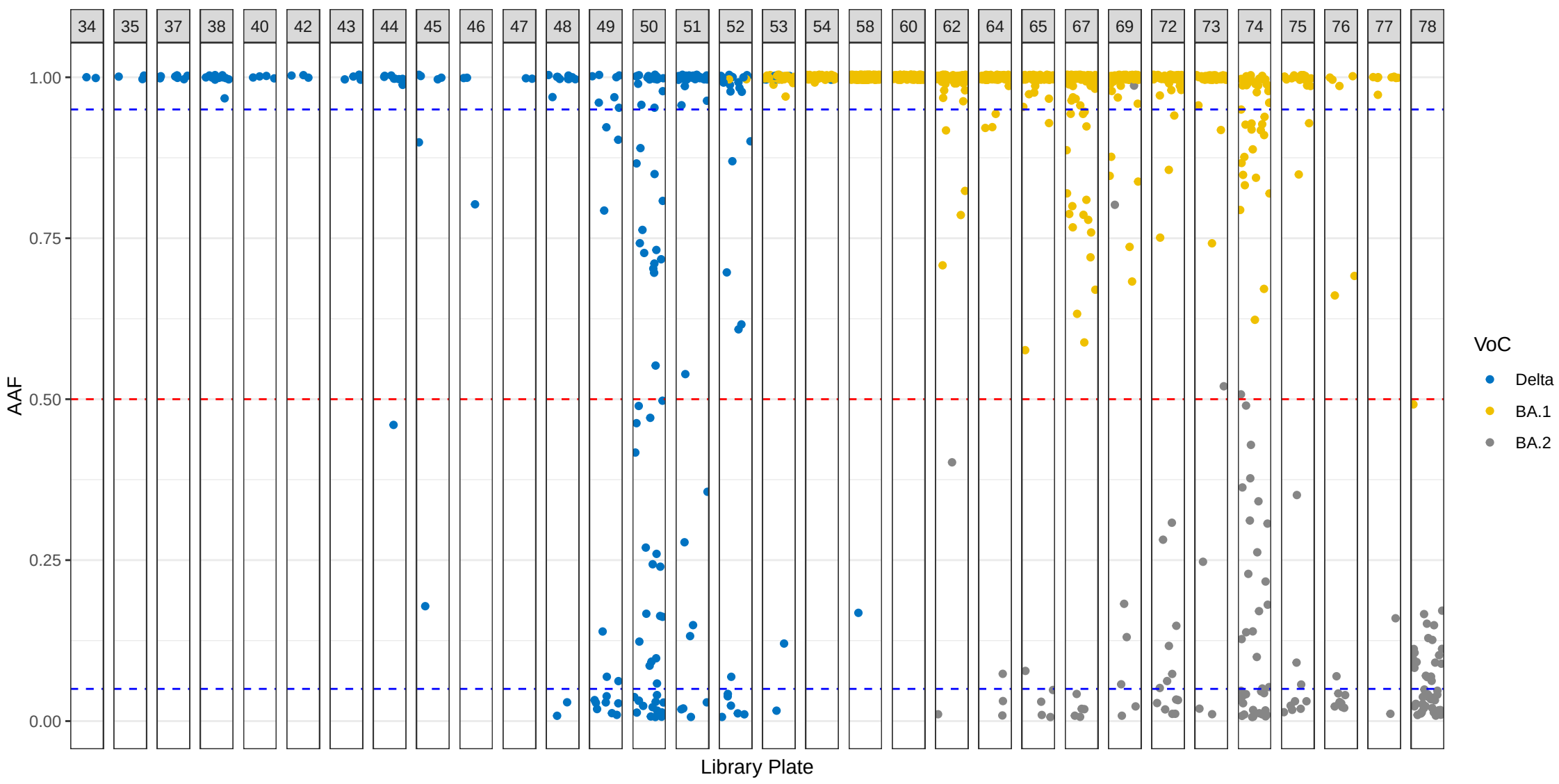

(c) Allele frequency (AF) of C21846T across sequencing batches.

Figure S4. C21846T - an example of well-to-well contamination in our dataset. C21846T is a BA.1-defining SNP, and is also present at consensus frequencies in some Delta sublineages. In our dataset, C21846T frequently appears as an iSNV in BA.2 isolates and Delta sublineages that do not typically carry the mutation, at minor allele frequency (AAF<0.5). The iSNV-carrying isolates are consistently found on plates with consensus-SNP carrying isolates ($\geq$0.5), suggesting that C21846T is likely a false positive iSNV caused by cross-sample contamination.

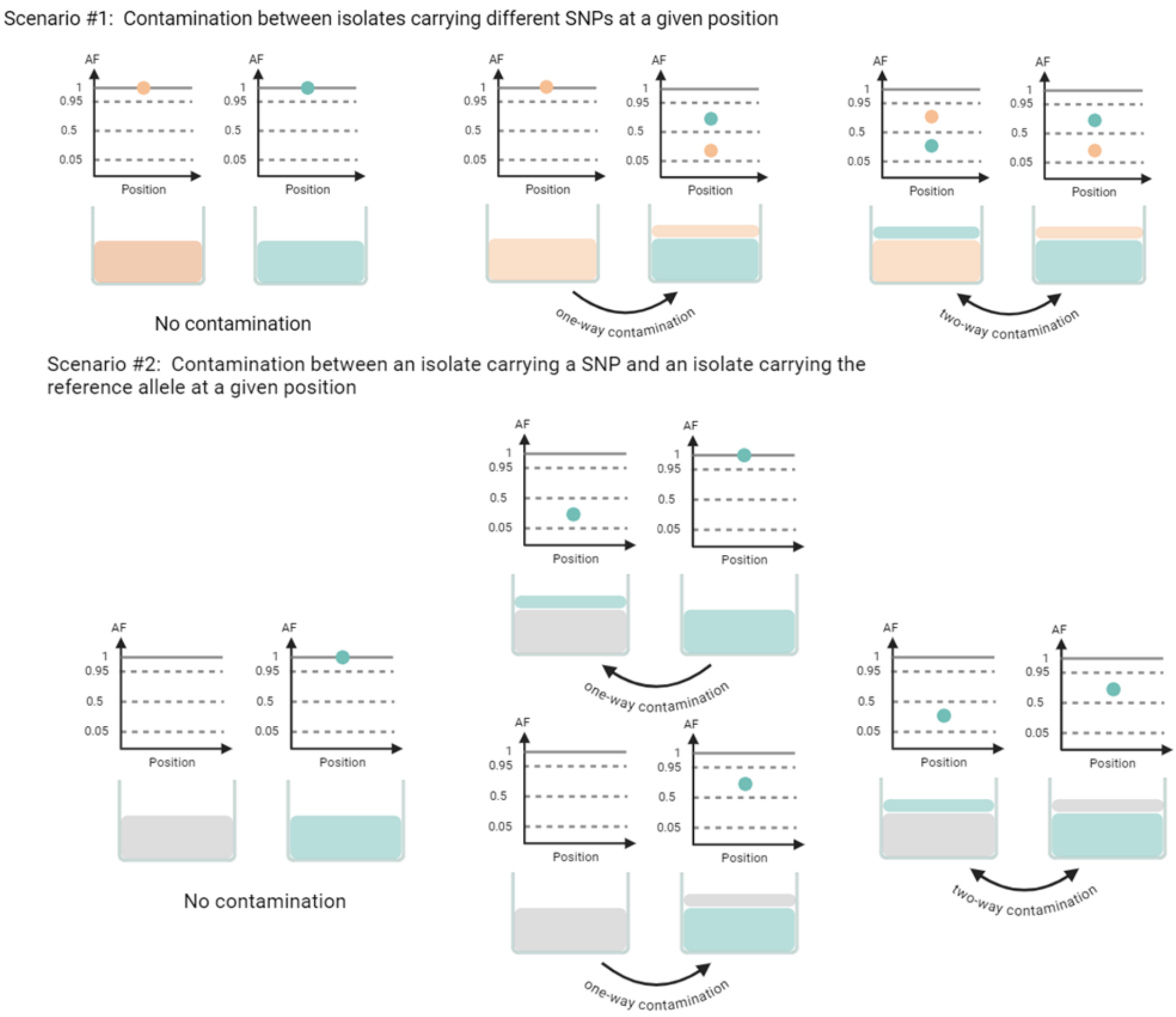

Figure S5. Illustration of Possible Contamination Scenarios.

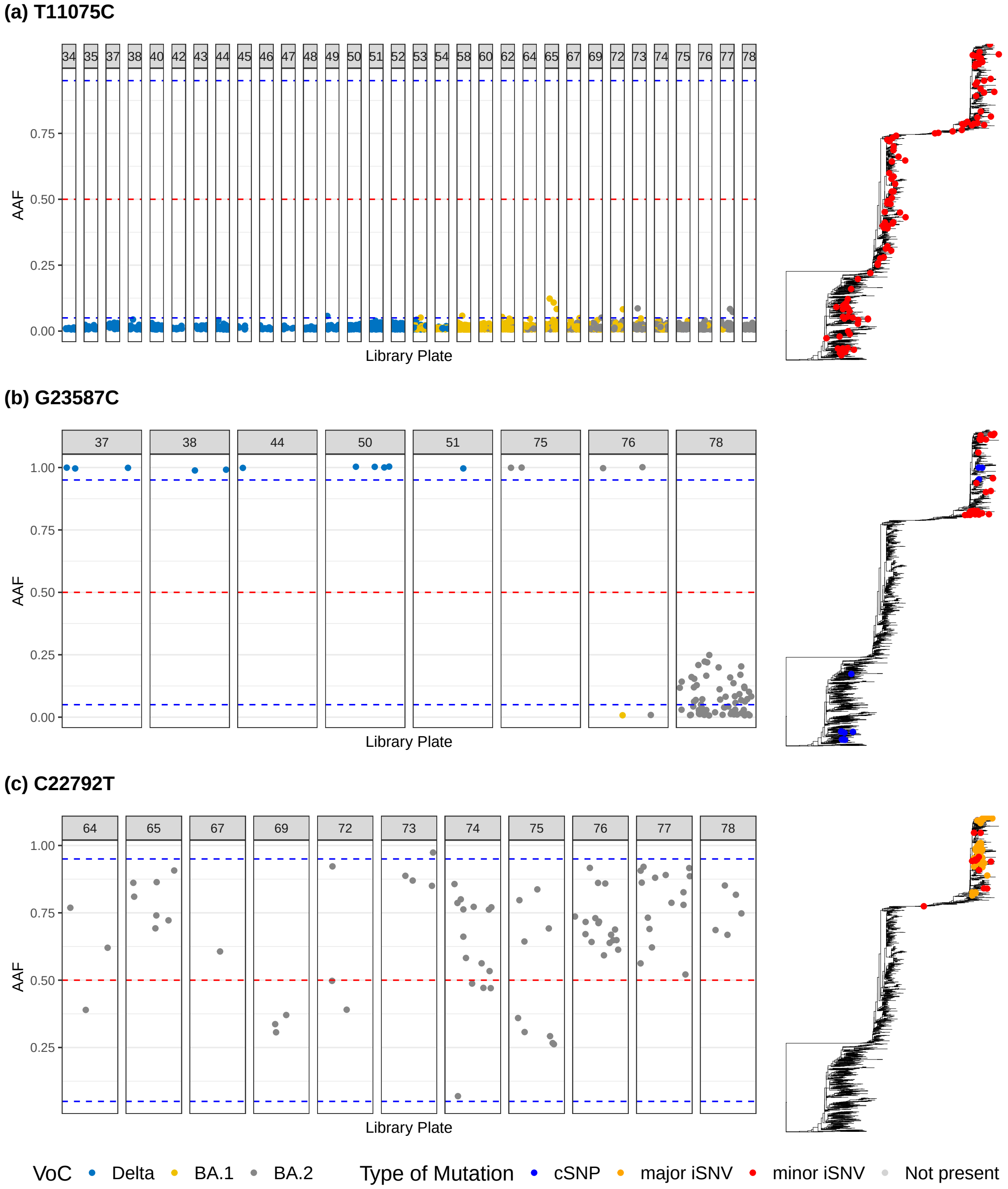

Figure S6. Examples of false-positive iSNVs from residual bias upon minimizing contamination. (a) T11075C is a previously reported residual systematic bias from sequencing artifact, appearing at low frequencies in many distantly-related isolates in our dataset (n = 2,138). [1] (b) G23587C represents likely residual contamination or artifact within a specific sequencing batch, recurrently detected at subconsensus frequencies in isolates (n = 63/130) processed on library plate 78. (c) C22792T is a known systematic artifact caused by failed primer binding. While it is a lineage-defining SNP for several BA.2 sublineages, its allele frequency remains consistently below the fixation threshold of 0.95.

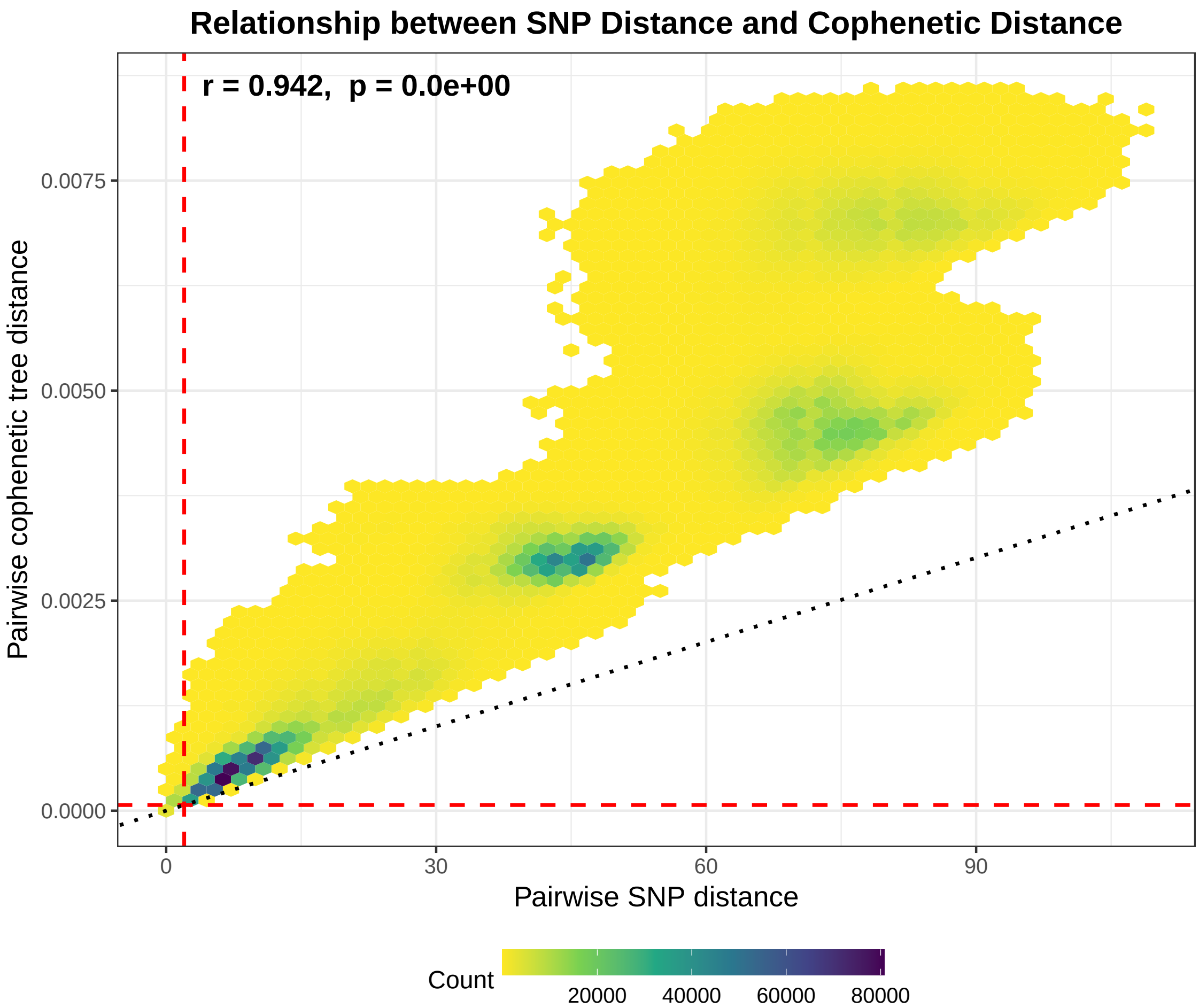

Figure S7. Correlation between SNP differences and patristic distances. SNP differences were counted from the multiple sequence alignment using SNP-dist [2]. Patristic distances were calculated from a maximum-likelihood phylogeny constructed with RAxML under the GTR+Gamma model, using the `cophenetic.phylo` function from the `ape` R package. Although the two measures are significantly correlated, SNP counts are systematically lower, likely because SNP differences do not account for site-specific variation in evolutionary rates and may therefore underestimate divergence times [3, 4]. Using cophenetic distances instead of SNP differences to identify potential transmission pairs is both more computationally intensive and more conservative, as indicated by the red dashed lines, but this approach makes fuller use of the sequence alignment.

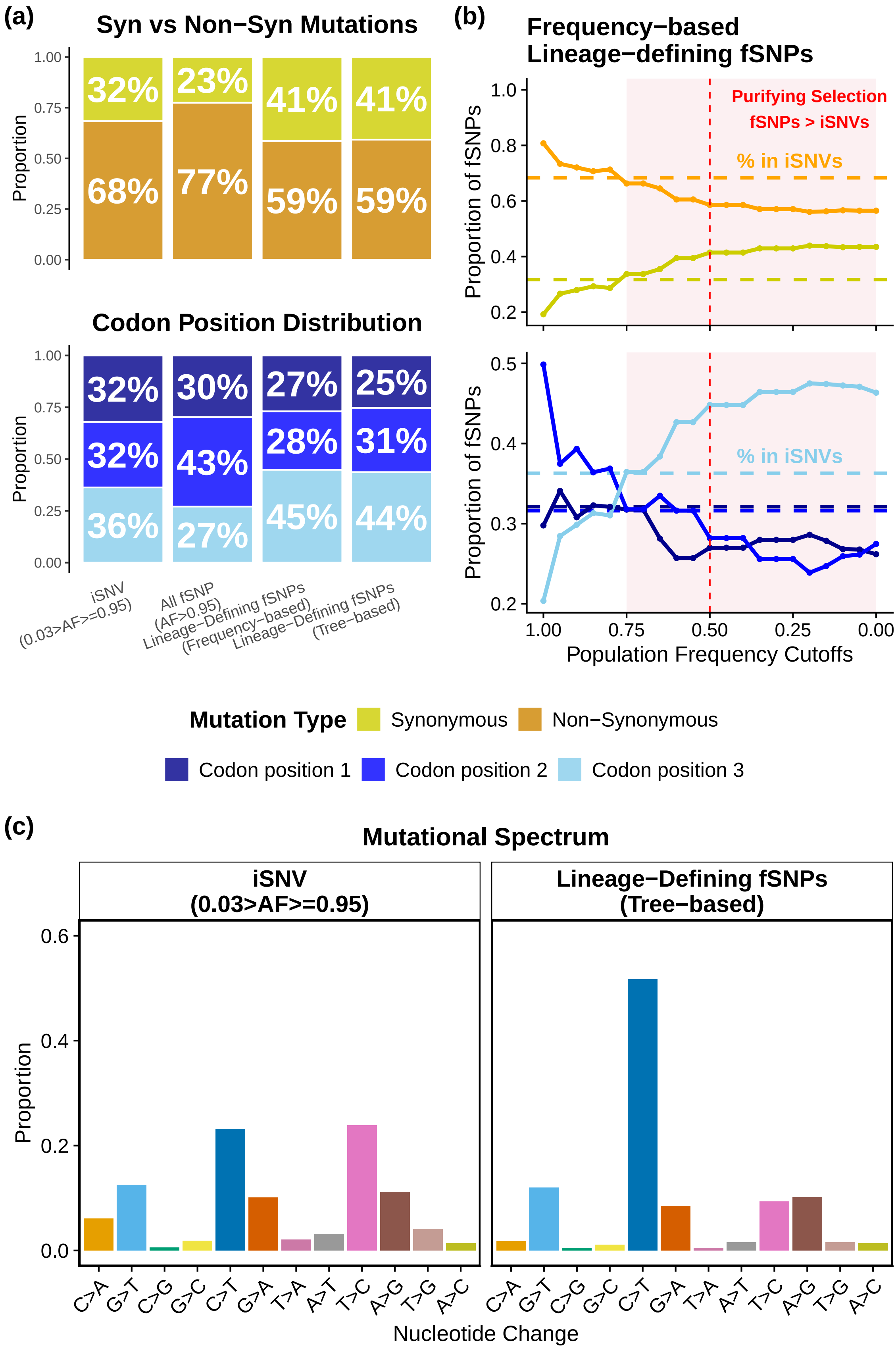
(a)
Syn vs Non−Syn Mutations
Proportion
32%
68%
23%
77%
41%
59%
41%
59%
Codon Position Distribution
32%
32%
36%
30%
43%
27%
27%
28%
45%
25%
31%
44%
iSNV (0.03>AF>=0.95)
All fSNP (AF>0.95)
Lineage−Defining fSNPs (Frequency−based)
Lineage−Defining fSNPs (Tree−based)
(b)
Frequency−based Lineage−defining fSNPs
Proportion of fSNPs
Purifying Selection
fSNPs > iSNVs
% in iSNVs
% in iSNVs
Population Frequency Cutoffs
Mutation Type
Synonymous
Non−Synonymous
Codon position 1
Codon position 2
Codon position 3
(c)
Mutational Spectrum
iSNV (0.03>AF>=0.95)
Lineage−Defining fSNPs (Tree−based)
Proportion
Nucleotide Change

Figure S8. Mutational Characteristics of iSNVs compared to fSNPs. (a) Proportion of synonymous and nonsynonymous mutations (top) and the distribution of mutations by codon position (bottom) among iSNVs ($3\% \leq \text{AAF} < 95\%$), all fixed SNPs (fSNPs, $\text{AAF} \geq 95\%$), and lineage-defining fixed SNPs. Counting all fixed SNPs across samples skews the mutational spectrum toward nonsynonymous and third-codon-position mutations, largely due to repeated counting of lineage-defining SNPs. To address this, lineage-defining SNPs can be counted only once, defined either by population frequency (frequency-based) or by mutation-annotated phylogenetic trees (tree-based). (b) Proportion of synonymous and nonsynonymous mutations (top) and distribution by codon position (bottom) across varying population frequency thresholds for defining frequency-based lineage-defining fSNPs. When the threshold is set at 75% or lower, the proportion of nonsynonymous and third-codon-position mutations among fSNPs falls below that of iSNVs, indicating stronger purifying selection. These proportions plateau at the 50% threshold, closely matching those obtained with the tree-based definition. (c) Mutational spectrum in iSNVs and tree-based lineage-defining fSNPs. Among fSNPs, we observe pronounced C>U over U>C and C>U over G>A asymmetries, consistent with the expected signature of host APOBEC-mediated cytidine deamination acting preferentially on the positive-sense viral RNA strand. In contrast, among iSNVs, we detect strong C>U over G>A and U>C over A>G asymmetries, indicating pronounced strand bias in mutation patterns but without a strong directional preference between C>U and U>C transitions. This pattern may reflect intrinsic features of the viral replication process, with iSNVs capturing the immediate outputs of viral polymerase errors or short-term editing activity, while fSNPs - having persisted over multiple replication and transmission cycles may reflect the cumulative effects of prolonged exposure to APOBEC-mediated editing.

Table S1. Categories and summary statistics for singleton and recurrent iSNVs across Delta, BA.1, and BA.2 lineages.

	Delta (n=777)	BA.1 (n=1,199)	BA.2 (n=794)	Overall (n=2,770)
**Singleton iSNVs**				
Total iSNVs	1,569	2,328	1,585	4,873
Non-coding	54	51	31	110
Synonymous (S)	434	743	494	1,481
Non-synonymous (N)	1,081	1,534	1,060	3,282
N/S	2.49	2.06	2.15	2.22
d_N/d_S (95% CI)	0.79 (0.70–0.90)	0.82 (0.74–0.90)	0.84 (0.75–0.95)	0.81 (0.76–0.87)
**Recurrent iSNVs**				
Total iSNVs	184	304	147	1,244
Non-coding	24	19	2	71
Synonymous (S)	34	108	66	398
Non-synonymous (N)	126	177	79	775
N/S	3.71	1.64	1.20	1.95
χ^2 p-value vs Singleton N/S	0.06	0.09	0.001	0.07
d_N/d_S (95% CI)	1.07 (0.71-1.61)	0.74 (0.57-0.96)	0.46 (0.32-0.66)	0.78 (0.68-0.89)
Total unique mutations	79	141	72	550
Non-coding	10	9	1	30
Synonymous (S)	16	50	32	173
Non-synonymous (N)	53	82	39	347
N/S	3.31	1.64	1.22	2.01
χ^2 p-value vs Singleton N/S	0.40	0.25	0.03	0.33
d_N/d_S (95% CI)	1.07 (0.58–1.96)	0.75 (0.51–1.09)	0.46 (0.27–0.78)	0.81 (0.67–0.99)

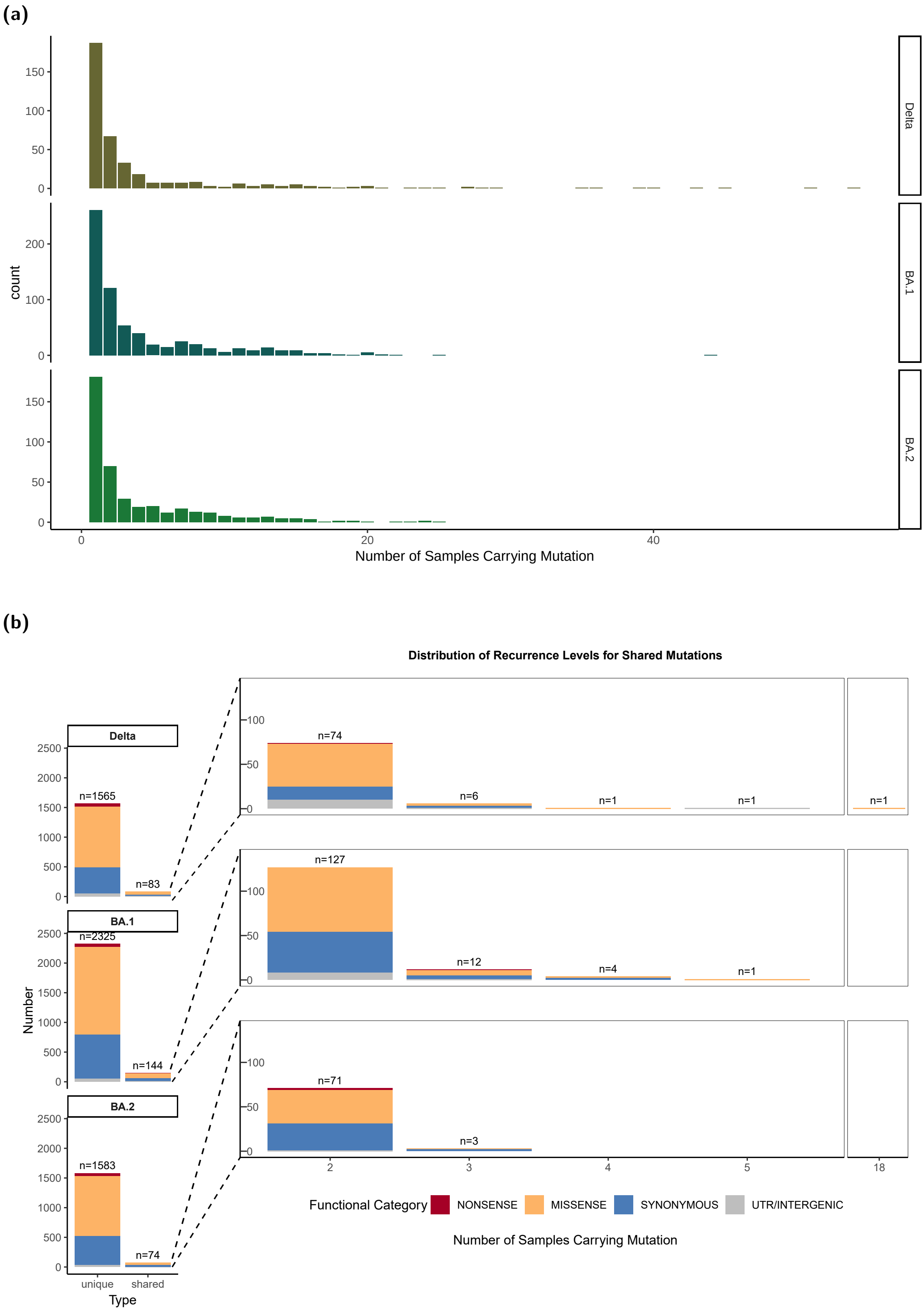

Figure S9. **(a) Distribution of the number of iSNVs per sample across VoCs**, and **(b) Counts of unique and shared iSNVs as well as their recurrence distribution across VoCs.** "Unique" mutations occur in a single sample, while "shared" mutations are found in multiple samples. The distribution of shared iSNVs by recurrence level (number of samples in which a shared iSNV appears) is also shown.

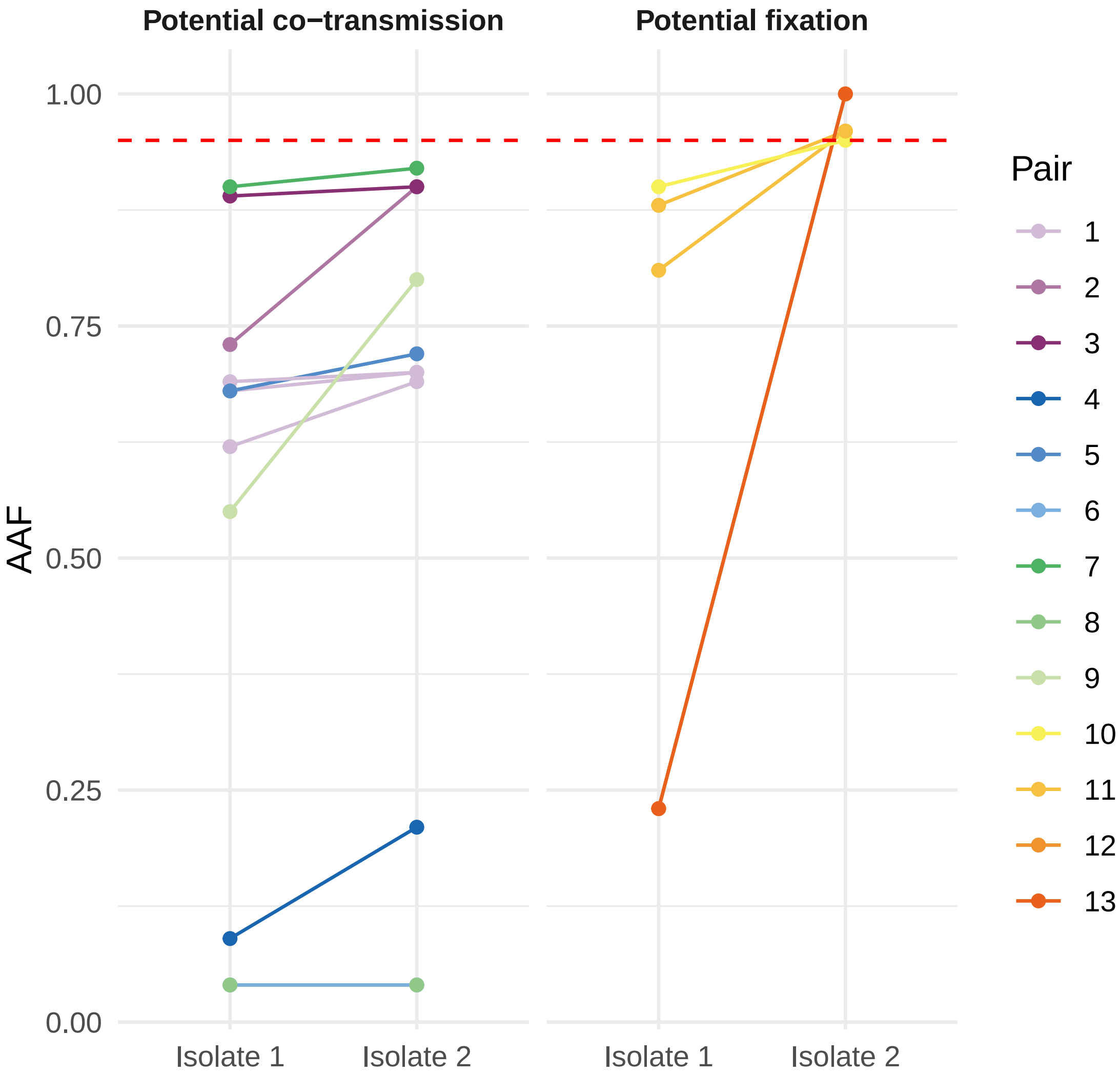

Figure S10. Allele frequencies of shared iSNVs in potential transmission pairs. Each line connects the alternative allele frequency (AAF) for a given iSNV between paired isolates. The left panel shows potential co-transmission events, where the iSNV is present in both isolates. The right panel shows potential fixation events, where the iSNV rises to fixation (AAF $\geq$0.95) in one of the isolates. Each pair is color-coded. The red dashed line marks the consensus threshold (AAF = 1).

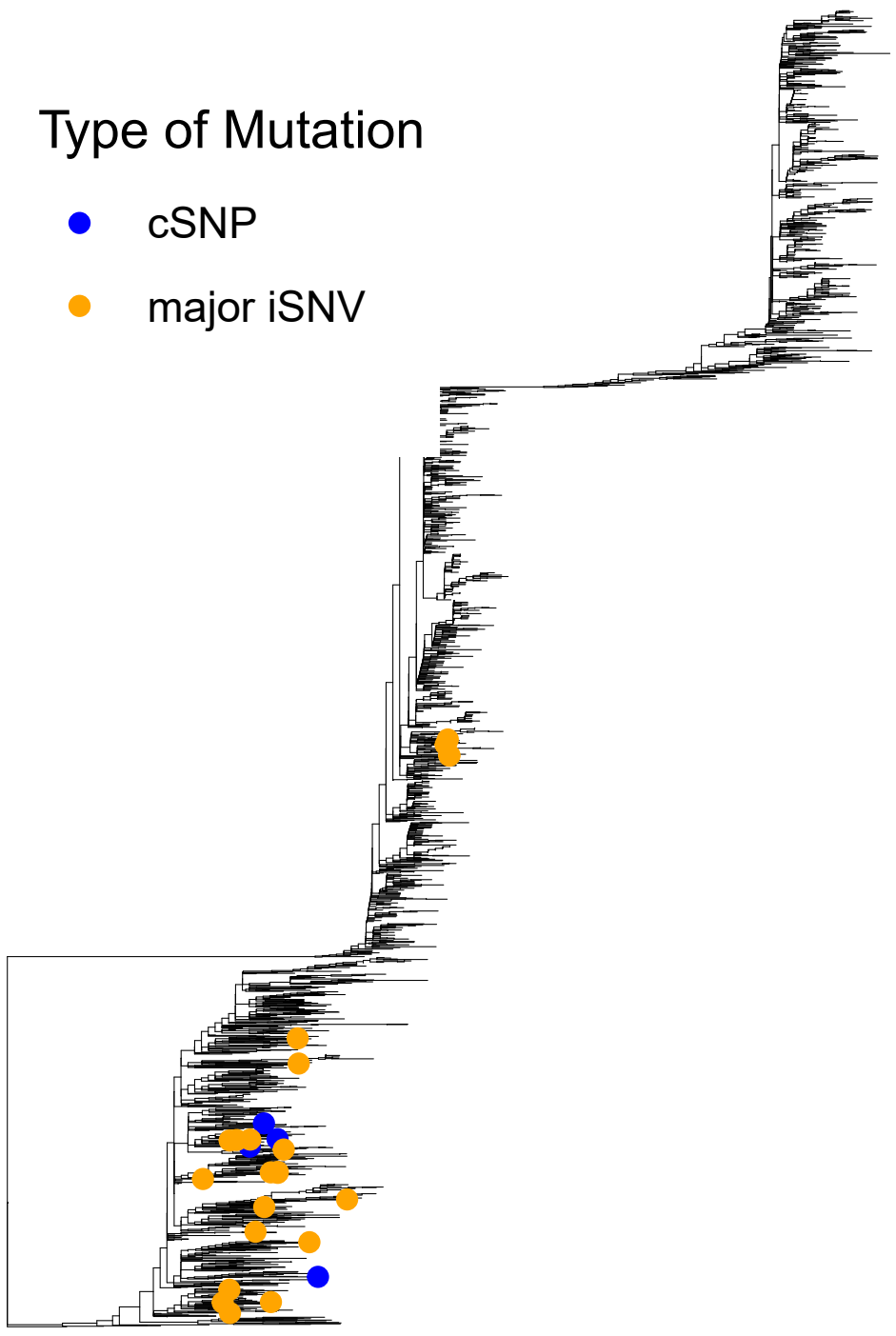

(a) Phylogenetic distribution of G11083T.

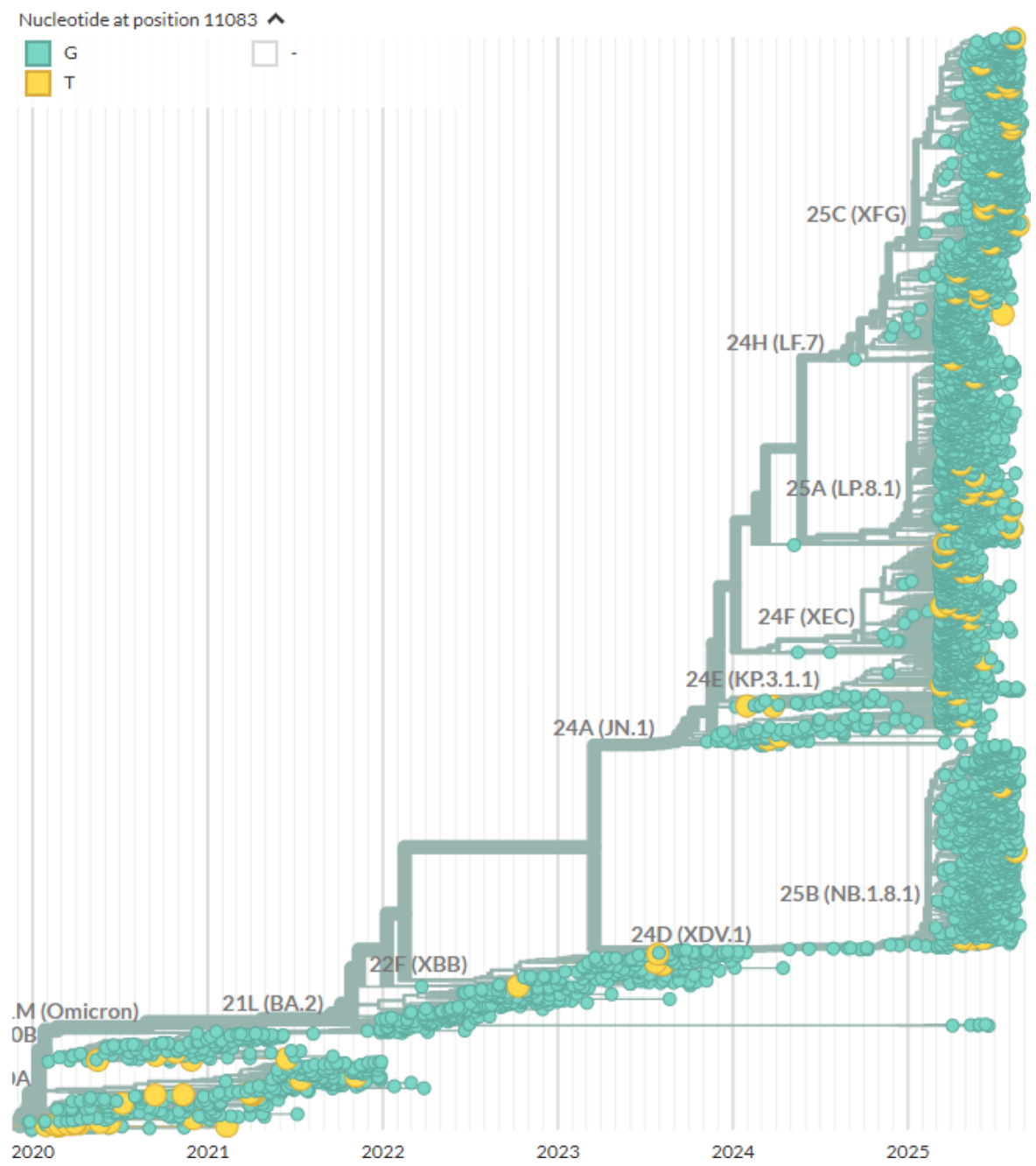

(b) Consensus allele at position 11,083 in GISAID (Nextstrain screenshot).

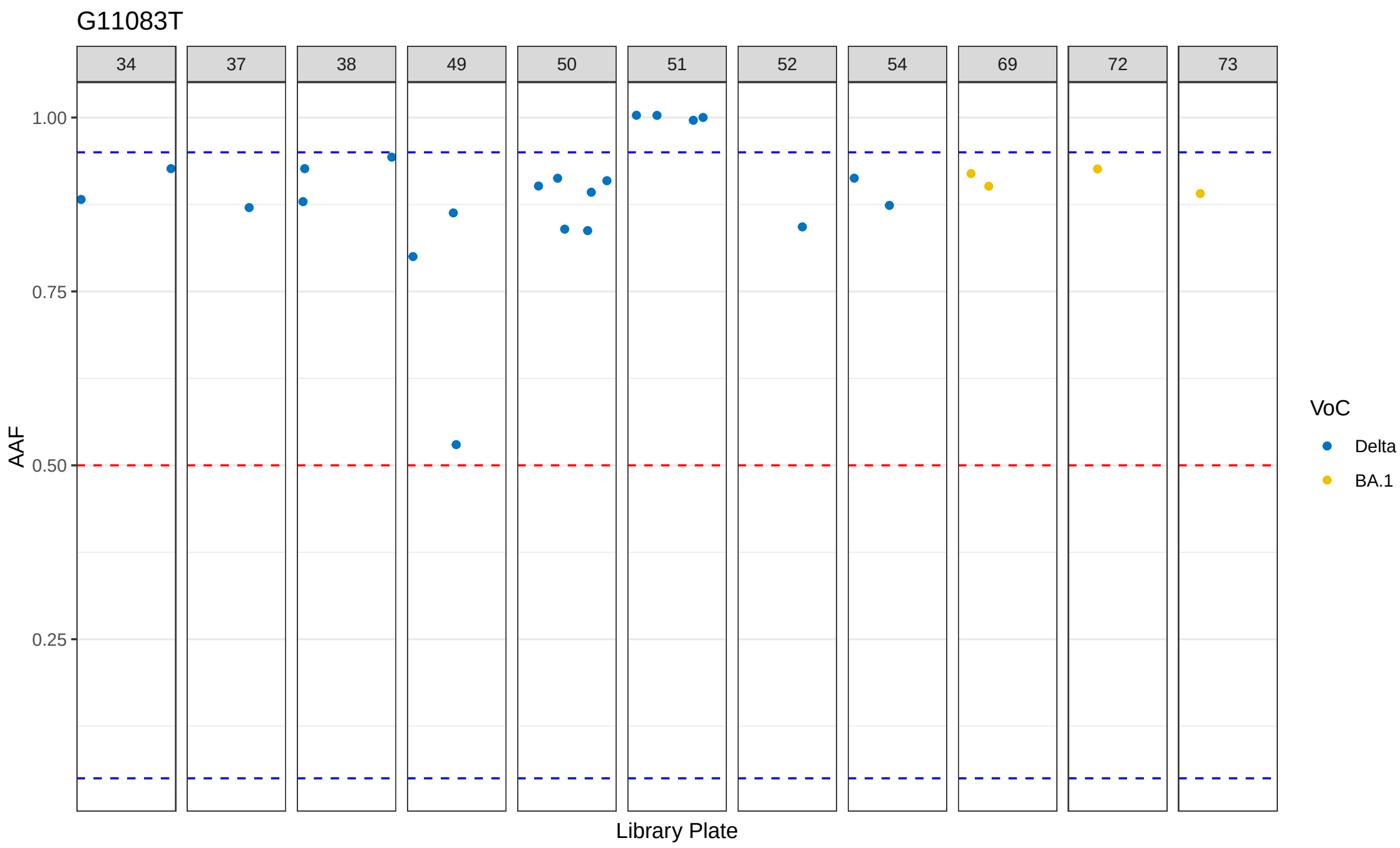

(c) Allele frequency (AF) of G11083T across sequencing batches.

Figure S11. G11083T - a highly recurrent mutation among iSNVs.

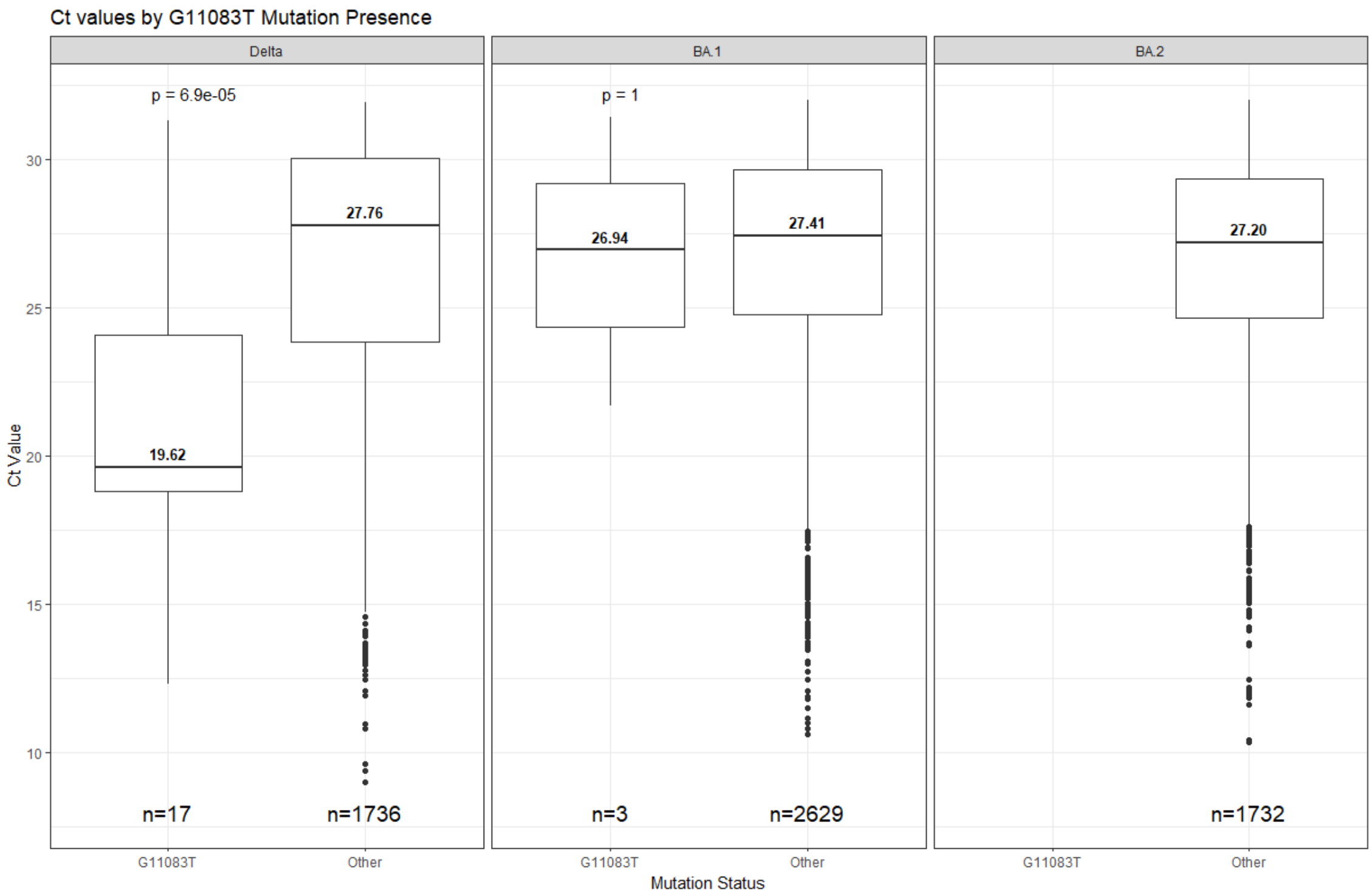

Figure S12. Distribution of Ct values for isolates with and without the iSNV G11083T.

References

[1] E. E. Bendall, D. Dimcheff, L. Papalambros, W. J. Fitzsimmons, Y. Zhu, J. Schmitz, N. Halasa, J. Chappell, E. T. Martin, J. E. Biddle, *et al.*, "In depth sequencing of a serially sampled household cohort reveals the within-host dynamics of omicron sars-cov-2 and rare selection of novel spike variants," *PLoS pathogens*, vol. 21, no. 4, p. e1013134, 2025.

[2] T. Seemann, "snp-dists: Pairwise snp distance matrix from a fasta sequence alignment." `https://github.com/tseemann/snp-dists`, 2018.

[3] A. F. Poon, "Impacts and shortcomings of genetic clustering methods for infectious disease outbreaks," *Virus evolution*, vol. 2, no. 2, p. vew031, 2016.

[4] B. Sobkowiak, K. Kamelian, J. E. Zlosnik, J. Tyson, A. G. d. Silva, L. M. Hoang, N. Prystajecky, and C. Colijn, "Cov2clusters: genomic clustering of sars-cov-2 sequences," *BMC genomics*, vol. 23, no. 1, p. 710, 2022.
